# Infrared Thermography Reveals Recurrent Thermal Asymmetry Patterns Rather than Stable Thermal Phenotypes in Elite Under-18 Soccer Players: A Prospective Longitudinal Observational Study

**DOI:** 10.3390/sports14090392

**Published:** 2026-09-04

**Authors:** Aldo Di Martino, Silvana Mirella Aliberti, Daria Nurzynska, Raffaele Canonico, Gennaro De Luca, Angelo Gioffredi, Clotilde Castaldo, Franca Di Meglio

**Affiliations:** 1 Department of Public Health, University of Naples “Federico II”, 80131 Naples, NA, Italy; aldo.dimartino@unina.it (A.D.M.); clotilde.castaldo@unina.it (C.C.); franca.dimeglio@unina.it (F.D.M.); 2 Department of Medicine, Surgery and Dentistry “Scuola Medica Salernitana”, University of Salerno, 84081 Baronissi, SA, Italy; dnurzynska@unisa.it; 3 Division of Preventive Medicine, Dresden International University (DIU), 01067 Dresden, Germany; 4 Unit of Dietetics, Sport Medicine and Psychophysical Wellbeing, Department of Experimental Medicine, University of Campania Luigi Vanvitelli, 80138 Naples, NA, Italy; 5 Società Sportiva Calcio Napoli, 80133 Naples, NA, Italy

**Keywords:** infrared thermography, skin temperature, soccer, youth athletes, injury prevention, lower-limb asymmetry, performance monitoring

## Abstract

Background: Evidence regarding the longitudinal use of infrared thermography in elite under-18 soccer players remains limited. Most previous studies have relied on cross-sectional designs or short observation periods. Methods: This prospective longitudinal observational study monitored 18 elite under-18 male outfield soccer players across 16 official matches (96 valid assessments). Thermographic evaluations were performed 48 h post-match under standardized conditions across six bilateral lower-limb regions. A thermal asymmetry was defined as relevant if ΔTsk ≥ 0.5 °C. Linear mixed-effects models, paired *t*-tests and chi-square tests were used for statistical analysis. Results: Relevant asymmetries were detected in 79.2% of assessments (mean maximum ΔTsk 0.77 ± 0.43 °C). Posterior Thigh BF (40.6%) and Posterior Thigh ST (35.4%) showed the highest prevalence (χ^2^ = 16.31, *p* = 0.006). Significant side-to-side differences were found for Anterior Thigh (*p* = 0.004), Adductor (*p* = 0.039) and Posterior Thigh BF (*p* = 0.032). The intraclass correlation coefficient for Thermal Asymmetry Burden was very low (ICC = 0.024). Fifteen athletes (83.3%) showed recurrent asymmetry and 13 (72.2%) showed a recurrent same-region pattern. Match-to-match variation in TAB was significant (Kruskal–Wallis *p* = 0.025; ANOVA *p* = 0.011). No time-loss muscle injuries occurred. Conclusions: Post-match thermal asymmetries are highly prevalent in elite youth soccer and appear to reflect dynamic, load-dependent recovery responses rather than stable thermal phenotypes. Longitudinal thermographic monitoring may help characterise individual recovery patterns when interpreted alongside other monitoring tools.

## 1. Introduction

Muscle injuries represent one of the most common medical issues in soccer, with a substantial impact on player availability, training continuity and team performance [1,2]. In elite settings, even brief periods of absence may affect both individual development and team competitiveness. For this reason, modern sports medicine has increasingly shifted from reactive treatment toward proactive monitoring, aiming to identify early indicators of muscle overload before they progress into clinically significant injuries [3,4].

Among the tools proposed for early detection of subclinical changes, infrared thermography has gained growing attention as non-invasive technique capable of rapidly assessing skin temperature distribution [5,6,7]. Variations in skin temperature may reflect local physiological responses such as altered blood flow, autonomic regulation, inflammatory processes, or muscle overload, making thermography particularly suitable for repeated assessments throughout the competitive season [5,6]. The rationale for its use in soccer relies on the observation that thermal asymmetries between contralateral regions may signal imbalances in tissue response to training and match demands. Previous studies in adult athletes suggest that asymmetries of approximately 0.4–0.5 °C may be clinically relevant, especially in muscle groups exposed to high mechanical stress [8,9]. The posterior thigh is of particular interest, as hamstring muscles are repeatedly loaded during sprinting, acceleration, deceleration and high-intensity running, making this region a key target for monitoring fatigue, overload and potential injury risk [1,2,10].

In recent years, the broader field of infrared thermography has advanced both physiologically and methodologically. Priego-Quesada and co-authors [11] have contributed to understanding skin temperature dynamics in relation to muscle metabolism, perfusion and mechanical load during exercise, providing a general physiological basis for interpreting thermographic responses. Brioschi’s [12] work has further emphasized the clinical meaning of thermal patterns in general thermographic assessment, supporting the integration of thermography within multimodal clinical frameworks. Within sport-specific contexts, Cabizosu and colleagues [13] examined lower-limb thermographic and morphological factors in relation to competitive performance in endurance athletes, contributing to refining acquisition protocols and the interpretation of thermal asymmetries in applied sport settings. Similarly, a recent study by Januário et al. [14] demonstrated excellent agreement between automated ThermoHuman^®^ analysis and manual ROI delineation before and after running, with concordance correlation coefficients ranging from 0.95 to 0.98 and mean absolute errors between 0.3 °C and 0.5 °C; automated processing also reduced analysis time by more than 85%, confirming the feasibility of standardized and time-efficient thermographic workflows in applied sport contexts. Collectively, these general and sport-specific contributions highlight the need to update the thermographic literature in soccer by incorporating recent evidence that strengthens both the physiological and methodological foundations of longitudinal monitoring.

Although thermography has been explored in adult professional soccer, where it has shown potential for guiding recovery strategies and supporting injury-prevention programs [8], the current body of evidence remains largely centred on senior players [9]. More recent research has examined the relationship between skin temperature, physical load and well-being status in elite soccer players, further supporting its role of thermography in load monitoring [10]. However, considerably less is known about its application in elite youth players, particularly under standardized and longitudinal monitoring conditions. This gap is clinically relevant because youth players are characterized by specific physiological and biomechanical characteristics related to growth and maturation, high-intensity training and progressive transition toward adult-level performance demands. In this context, subclinical muscle load patterns may not always manifest as symptoms, making early detection tools especially valuable.

Evidence from tennis, padel and combat sports consistently report that asymmetries may reflect sport-specific adaptations rather than pathology, supporting the need for athlete-specific interpretation rather than rigid thresholds [15,16,17]. Accordingly, a further limitation of the literature is that thermographic findings are often reported without clear explanation of how they inform clinical decision-making. In practice, clinicians may face isolated thermal findings without structured guidance on how to integrate them into daily load-management strategies. The value of thermography may therefore depend not only on detecting asymmetries but also on applying predefined thresholds within a standardized interventions protocol. Moreover, emerging evidence suggests that repeated thermographic assessments may reveal not only acute asymmetries but also individual patterns of thermal response, potentially reflecting athlete-specific adaptations to load [9]. However, this aspect remains largely unexplored in youth soccer.

To date, evidence regarding the longitudinal use of infrared thermography in elite under-18 soccer players within real-world clinical environments remains limited, with most published studies relying on cross-sectional designs, short observation periods, or heterogeneous monitoring protocols [5,18,19]. Specifically, there is a lack of data describing the prevalence and anatomical distribution of post-match thermal asymmetries in youth players monitored longitudinally under controlled acquisition conditions and managed through a predefined thermography-guided intervention protocol. This represents the main scientific gap addressed by the present study.

Therefore, the primary aim of this study was to describe the prevalence and anatomical distribution of thermal asymmetries in an elite under-18 soccer team monitored 48 h after official matches under standardized conditions. Secondary aims were to investigate the recurrence of thermal asymmetries within individual athletes, characterize recurrent athlete-specific thermal asymmetry patterns, and explore the longitudinal evolution of thermal asymmetry burden throughout the competitive season within a structured thermography-guided intervention program.

### Research Hypothesis

Based on previous evidence and recent advances in thermographic interpretation, we hypothesized that:

1. Thermal asymmetries would be frequently detectable 48 h post-match, even in the absence of symptoms.
2. The posterior thigh would show the highest prevalence of asymmetries, reflecting its biomechanical load profile.
3. Individual athletes would exhibit recurrent and region-specific thermal patterns across repeated assessments.

## 2. Materials and Methods

### 2.1. Study Design and Participants

This prospective longitudinal observational study employed a repeated-measures design and was conducted in an elite under-18 soccer team during the competitive season. Eighteen male outfield players aged 17–18 years were monitored across 16 official matches between September 2025 and March 2026. Where an opponent was faced twice during the season under the league’s home-and-away format, the return fixture is identified in the tables and figures by suffix ‘R’. Playing positions included defenders, midfielders and forwards.

**Figure 1:**
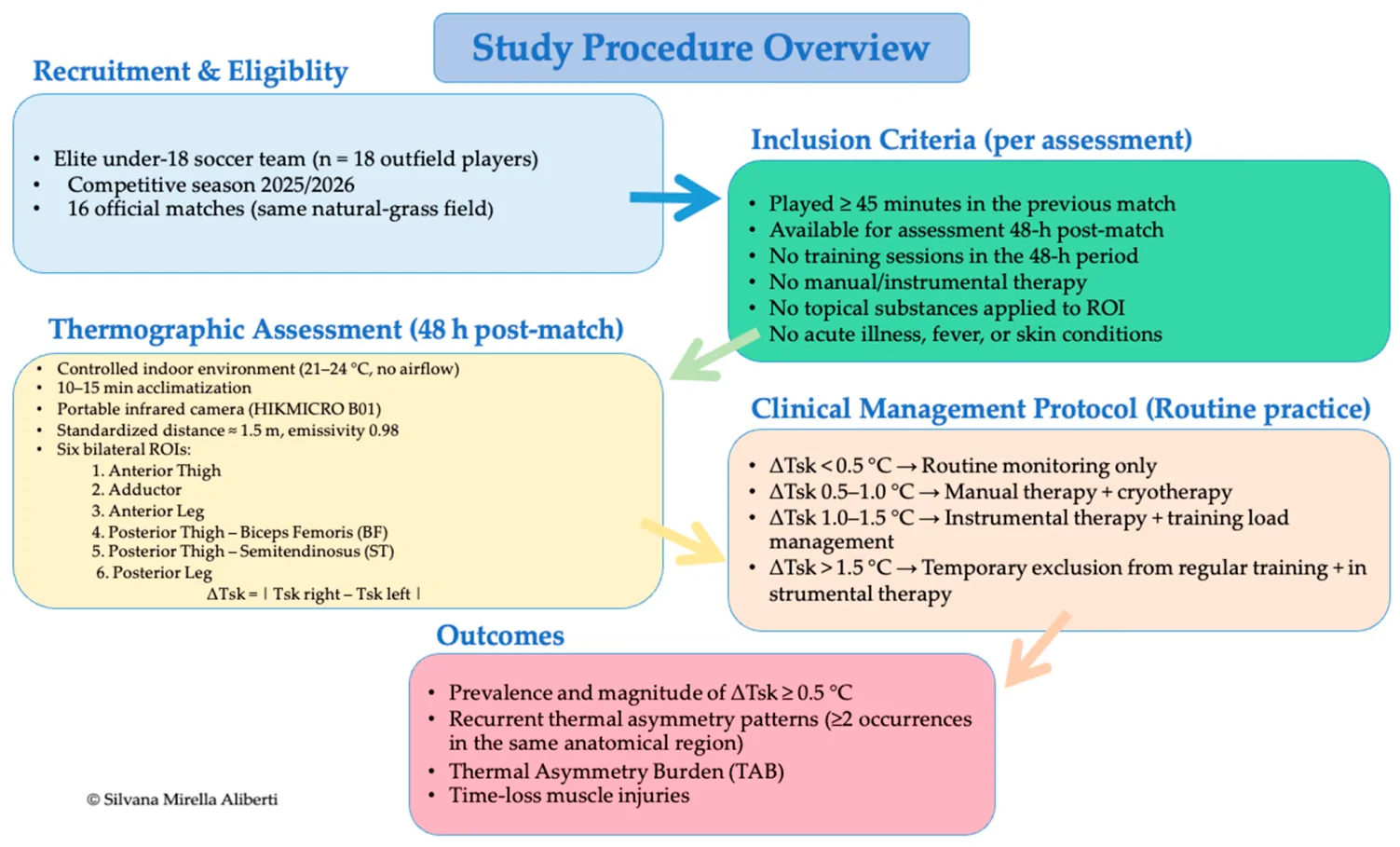
Schematic overview of the study procedure, including prospective longitudinal thermographic monitoring, assessment eligibility, standardized 48-h post-match thermographic evaluation, routine clinical management thresholds, and study outcomes in elite under-18 soccer players. ROI = Region of Interest.

Players were eligible for inclusion in each post-match assessment if they had participated for at least 45 min in the preceding official match and were available for thermographic evaluation 48 h after match completion. The number of valid assessments per athlete ranged from 1 to 15 (mean 5.33 ± 3.65, median 5). Not all players were assessed after every match because of the predefined inclusion criteria (≥45 min of playing time and availability for thermographic assessment 48 h post-match). The unequal number of observations contributed by each athlete reflects the repeated-measures observational design and the predefined assessment eligibility criteria. The overall study procedure is summarized in Figure 1.

Thermographic assessments were performed only in athletes who completed at least 48 h of rest after match participation. During this period, players did not perform additional training sessions and did not receive manual or instrumental therapeutic interventions. Athletes were also instructed not to apply creams, gels or topical substances to the regions of interest before the assessment, in order to avoid potential interference with skin temperature measurements.

Players were excluded from individual assessments if they had performed training or recovery sessions during the 48-h period, received manual or instrumental treatment, applied topical substances to the examined regions, or presented acute illness, fever or skin conditions that could affect thermographic readings.

This study was conducted in accordance with the ethical principles of the Declaration of Helsinki and with current European and Italian regulations on the processing of health-related data for research purposes. The thermographic assessments were performed as part of routine clinical monitoring and did not involve any additional procedures beyond standard care. According to the European General Data Protection Regulation (Regulation EU 2016/679–GDPR), the Italian Legislative Decree 101/2018, and the AIFA guidelines for observational non-interventional studies (2017), this project qualifies as a non-interventional observational study. As such, it does not require formal approval by an Ethics Committee nor the assignment of an ethics protocol number.

All data were anonymized prior to analysis by removing personal identifiers and assigning alphanumeric codes to each athlete. Written informed consent for the use of anonymized data for research purposes was obtained from all athletes and, for minors, from their parents or legal guardians. Data were stored on a secure, access-restricted server in compliance with GDPR requirements for the protection of sensitive information.

### 2.2. Thermographic Assessment

Thermographic assessments were performed 48 h post-match, corresponding to the recovery phase of the weekly microcycle. The 48-h time frame was selected to ensure that thermographic measurements reflected subclinical muscle overload rather than acute post-exercise responses. Short-term skin temperature changes within the first 24 h are influenced by transient thermophysiological processes—such as vasodilation, inflammation and pain-related mechanisms—that reduce the stability and interpretability of early post-exercise thermographic readings [20]. The 48-h window represents a more stable and standardized phase of post-match recovery in elite soccer, allowing thermal asymmetries to be evaluated with greater physiological consistency and clinical relevance within longitudinal monitoring protocols.

Infrared images were acquired using a portable thermal camera (HIKMICRO B01, Hikmicro, Hangzhou, China), with a thermal sensitivity of <40 mK and an infrared resolution of 256 × 192 pixels, enhanced to 320 × 240 with SuperIR technology.

All measurements were conducted under standardized environmental and acquisition conditions. All assessments took place in the same indoor room within the club’s medical facility, ensuring full control of environmental variables. The room maintained a constant temperature of 21–24 °C, stable humidity, absence of direct sunlight, and no airflow from windows or ventilation systems. Atmospheric pressure and humidity did not vary meaningfully across sessions due to the indoor setting. To ensure consistency in post-match recovery conditions, all thermographic assessments were performed in the same indoor facility under standardized environmental conditions, irrespective of whether the preceding match was played at home or away.

Before image acquisition, athletes underwent a 10–15-min acclimatization period inside the controlled environment. Emissivity was set at 0.98 for human skin, and images were captured at a standardized distance of approximately 1.5 m. All assessments were performed by the same operator to reduce inter-observer variability.

Participants were assessed in a standing position, wearing minimal clothing to expose the lower-limb regions of interest. For anterior acquisitions, players stood upright with feet shoulder-width apart and the lower limbs fully visible. For posterior acquisitions, players maintained the same stance while facing away from the camera. The camera was positioned perpendicular to the anatomical region being assessed, and images were acquired in anterior and posterior views.

Six bilateral lower-limb regions of interest (ROI) were analyzed: anterior thigh, adductor region, anterior leg, posterior thigh corresponding to the biceps femoris region (BF), posterior thigh corresponding to the semitendinosus region (ST) and posterior leg. ROI placement was standardized using anatomical landmarks and kept consistent across repeated assessments. For each ROI, the mean skin temperature was recorded on both the right and left sides. Skin temperature asymmetry (ΔTsk) was defined as the absolute temperature difference between contralateral regions, calculated as:

ΔTsk = |Tsk _right ROI_ − Tsk _left ROI_|

Higher ΔTsk values indicated greater bilateral thermal asymmetry, independently of the warmer side.

### 2.3. Intervention Protocol

A structured, threshold-based intervention protocol was applied according to the magnitude of thermal asymmetry. The operational threshold for a relevant asymmetry was defined as Delta Tsk ≥0.5 °C and was used to guide clinical decision-making within the weekly training microcycle.

For Delta Tsk values < 0.5 °C, athletes underwent routine monitoring only, without specific intervention. For Delta Tsk values between 0.5 °C and 1.0 °C, manual therapy and cryotherapy were applied. Cryotherapy was performed using Game Ready compression cold therapy when available, and treatment duration was approximately 20 min per athlete. For Delta Tsk values between 1.0 °C and 1.5 °C, instrumental therapy and training load management were implemented. Instrumental therapy included laser therapy, TECAR therapy, ultrasound therapy, magnetotherapy or Game Ready treatment according to clinical evaluation, affected region and resource availability. Training load management consisted of reducing or modifying training exposure according to the assessment of the medical and performance staff. Treatment duration was approximately 20 min per athlete. For Delta Tsk values > 1.5 °C, the athlete was temporarily withheld from regular training and underwent instrumental therapy. Instrumental treatments included laser therapy, TECAR therapy, ultrasound therapy, magnetotherapy or Game Ready treatment according to the affected region and clinical judgement. Return to full training was considered after clinical reassessment and improvement of the thermographic abnormality.

All interventions were individualized and integrated into the weekly training program in collaboration with the medical and performance staff.

### 2.4. Outcome Measures

The primary outcomes were the prevalence and magnitude of thermal asymmetries exceeding the predefined ΔTsk threshold of 0.5 °C (ΔTsk ≥ 0.5 °C). Additional exploratory outcomes included: (i) recurrence of thermal asymmetries ≥ 0.5 °C within individual athletes; (ii) identification of athlete-specific recurrent thermal asymmetry patterns, defined as the anatomical region most frequently exceeding the ΔTsk ≥ 0.5 °C threshold across repeated assessments; and (iii) longitudinal variation in Thermal Asymmetry Burden (TAB) throughout the season.

For the purpose of this study, the following operational definitions were applied. A relevant thermal asymmetry was defined as ΔTsk ≥ 0.5 °C in any region of interest at a single assessment. An athlete was classified as showing recurrent asymmetry if this threshold was exceeded in at least two different assessments, irrespective of the anatomical region involved. A recurrent same-region asymmetry pattern was defined as the same anatomical region exceeding the ΔTsk ≥ 0.5 °C threshold on at least two assessments. For athletes with three or more valid assessments, the most frequently abnormal region was identified and the proportion of assessments in which that region was abnormal was calculated as a measure of consistency.

The operational threshold of ΔTsk ≥ 0.5 °C was selected because it represents the value most consistently reported in adult sports-thermography literature as a conventional cutoff [8,9,18,19]. However, because no age-specific validated threshold currently exists for elite under-18 soccer players, this value was adopted as a predefined reference threshold rather than an established clinical biomarker. Consequently, asymmetries exceeding this threshold are described herein as predefined threshold exceedances or relevant thermal asymmetries rather than definitively ‘clinically relevant’ asymmetries. Although a range of 0.4–0.5 °C is sometimes mentioned, 0.5 °C remains the conventional cut-off adopted in soccer studies and in the Delphi consensus on skin-temperature measurement [5]. The thermal camera used in the present study (HIKMICRO B01) has a thermal sensitivity < 40 mK (0.04 °C), well below this threshold, ensuring adequate measurement resolution.

To characterize the overall magnitude of lower-limb thermal asymmetry at each assessment, a Thermal Asymmetry Burden (*TAB*) score was calculated as the mean ΔTsk across the six predefined regions of interest:

$$TAB=\frac{\left(Anterior\;Thigh+Adductor+Anterior\;Leg+Posterior\;Thigh_{BF}+Posterior\;Thigh_{ST}+Posterior\;Leg\right)}{6}$$

Higher TAB values indicate a greater overall burden of bilateral thermal asymmetry across the lower limbs. In addition, the maximum thermal asymmetry (ΔTsk) was recorded for each assessment. Regional prevalence was calculated as the proportion of assessments in which each ROI showed ΔTsk ≥ 0.5 °C.

The secondary outcome was the occurrence of time-loss muscle injuries during the observation period. Muscle injury was defined as any clinically diagnosed muscle injury resulting in absence from training or match participation.

### 2.5. Statistical Analysis

Descriptive statistics were used to summarize continuous variables (mean ± standard deviation, median and interquartile range) and categorical variables (frequencies and percentages).

Because each athlete contributed multiple assessments, the hierarchical (repeated-measures) structure of the data was accounted for by linear mixed-effects models with a random intercept for athlete. The primary continuous outcomes (Thermal Asymmetry Burden—TAB, and maximum ΔTsk per assessment) were analysed with mixed models of the form:

Outcome ~ fixed effects + (1 | Athlete)

Intraclass correlation coefficients (ICC) were calculated from the variance components of the mixed models to quantify the proportion of variance attributable to between-athlete differences. When the between-athlete variance component approached the boundary of the parameter space, generalized estimating equations (GEE) with an exchangeable working correlation structure and robust standard errors were used as a complementary approach.

Differences between contralateral limbs (Right vs. Left) for each of the six regions of interest were examined with three complementary approaches to account for the repeated-measures structure: (i) conventional paired Student’s *t*-tests treating all 96 assessments as independent observations; (ii) paired *t*-tests performed on athlete-level mean values (one value per athlete, n = 18); (iii) generalized estimating equations (GEE) with an exchangeable working correlation structure and robust standard errors. Linear mixed-effects models with a random intercept for athlete were also explored; however, because the between-athlete variance component approached the boundary of the parameter space, GEE was adopted as the primary clustering-adjusted approach.

Match-to-match variation in TAB was assessed with a Kruskal–Wallis test and one-way ANOVA. The non-parametric Kruskal–Wallis test was used as the primary test, given the skewed distribution of TAB values; one-way ANOVA was additionally reported as a parametric complement, to verify that the finding was not dependent on the choice of test. The mixed-model framework was also used to confirm differences after accounting for the repeated-measures structure. To account for clustering within athletes, the same comparison was repeated using generalized estimating equations (GEE) with an exchangeable working correlation; an omnibus Wald test was used to evaluate the overall effect of Match.

The prevalence of relevant thermal asymmetries (ΔTsk ≥ 0.5 °C) across the six anatomical regions was compared with a chi-square goodness-of-fit test.

To evaluate the consistency of region-specific patterns, the most frequent region exceeding the 0.5 °C threshold was identified for each athlete with ≥3 assessments, and the proportion of assessments in which that region was abnormal was calculated. Athletes who showed the same region ≥ 0.5 °C on at least two occasions were classified as exhibiting a recurrent same-region pattern. Recurrent asymmetry (any region) was defined as the presence of a thermal asymmetry of ΔTsk ≥ 0.5 °C in at least two assessments.

No other inferential statistical analysis was performed beyond those described above. All analyses were conducted using STATA 16.1 (StataCorp MP: College Station, TX, USA, 2019) and Microsoft Excel (Microsoft Corp., Redmond, WA, USA). A two-tailed *p*-value < 0.05 was considered statistically significant.

## 3. Results

Eighteen elite under-18 male outfield soccer players were monitored across 16 official matches during the 2025/2026 competitive season, resulting in a total of 96 valid thermographic assessments performed 48 h post-match (Table 1).

The number of assessments per athlete ranged from 1 to 15 (mean 5.33 ± 3.65, median 5). One athlete was assessed only once, while three athletes contributed 10 or more assessments (Table 1a,b).

Thermal asymmetries exceeding the predefined threshold of ΔTsk ≥ 0.5 °C in at least one region were detected in 76 out of 96 assessments (79.2%). The mean maximum ΔTsk per assessment was 0.77 ± 0.43 °C, with a median of 0.70 °C (IQR 0.50–1.00 °C) and range 0.20–2.40 °C. Fifteen of the 18 athletes (83.3%) exhibited recurrent asymmetry (relevant asymmetry present in at least two assessments). Thirteen athletes (72.2%) showed a recurrent same-region asymmetry pattern (the same anatomical region exceeding 0.5 °C on at least two occasions) (Table 2).

When assessments were classified according to the highest ΔTsk value recorded, 20 assessments (20.8%) had a maximum ΔTsk < 0.5 °C, 49 assessments (51.0%) showed maximum ΔTsk values between 0.5 °C and <1.0 °C, 22 assessments (22.9%) showed values between 1.0 °C and 1.5 °C, and 5 assessments (5.2%) showed values > 1.5 °C (Table 2).

The linear mixed-effects model for TAB yielded an intercept (overall mean) of 0.358 °C (SE 0.019, *p* < 0.001). Between-athlete variance was 0.00065 and residual (within-athlete) variance was 0.02604, corresponding to an intraclass correlation coefficient (ICC) of 0.024. The very low ICC indicates that only 2.4% of the total variance was attributable to stable between-athlete differences.

When all 96 assessments were treated as independent, paired Student’s *t*-tests indicated significant side-to-side differences for Anterior Thigh (mean difference +0.12 °C, *p* = 0.004), Adductor (mean difference −0.09 °C, *p* = 0.039) and Posterior Thigh BF (mean difference −0.14 °C, *p* = 0.032). After accounting for clustering within athletes, only the Anterior Thigh difference remained consistently significant across approaches (athlete-level *p* = 0.049; GEE *p* = 0.002). The Adductor and Posterior Thigh BF differences were no longer statistically significant (athlete-level *p* = 0.233 and 0.132; GEE *p* = 0.072 and 0.060, respectively). Anterior Leg reached significance only under the GEE approach (*p* = 0.024) (Table 3).

**Figure 2:**
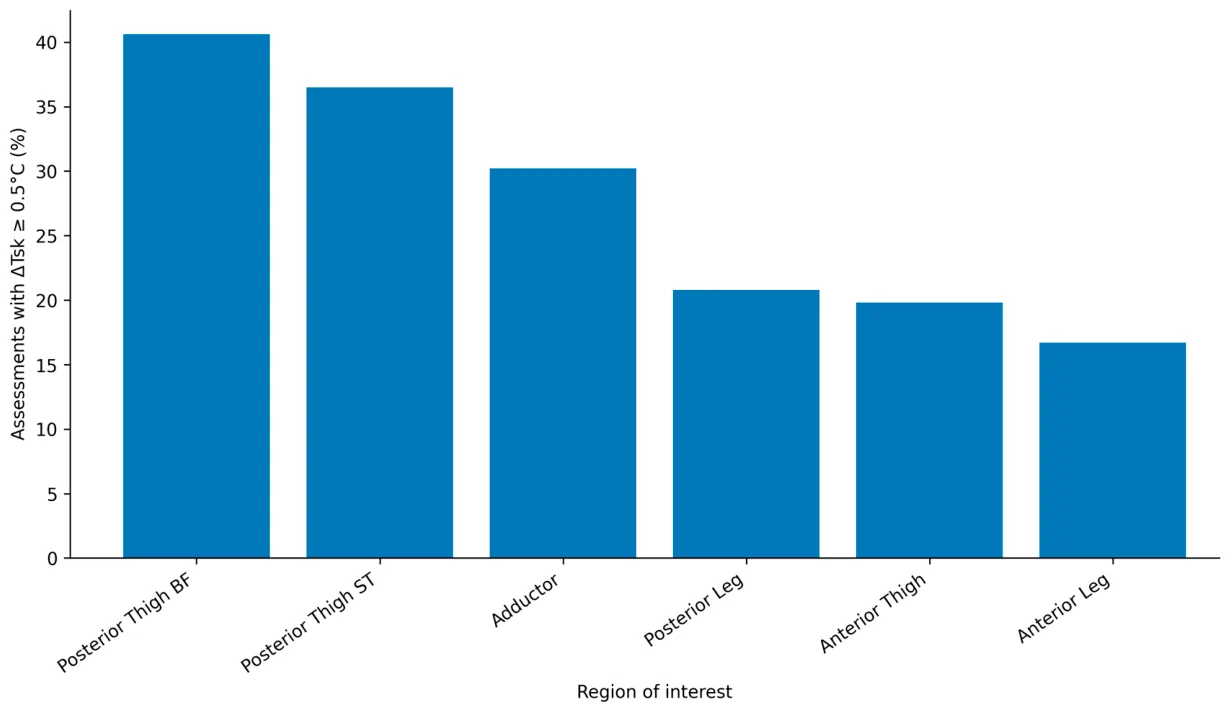
Prevalence of relevant thermal asymmetries (ΔTsk ≥ 0.5 °C) across the six lower-limb regions of interest in 96 post-match assessments.

The anatomical distribution of thermal asymmetries exceeding the predefined threshold of ΔTsk ≥ 0.5 °C showed a clear predominance of the posterior thigh regions (Table 4). Prevalence differed significantly among the six regions of interest (χ^2^ = 16.31, *p* = 0.006). Posterior Thigh BF exceeded the ≥ 0.5 °C threshold in 39 of 96 assessments (40.6%), followed by Posterior Thigh ST (34/96, 35.4%) and Adductor (29/96, 30.2%) (Figure 2).

At the individual level, 15 out of 18 athletes (83.3%) presented thermal asymmetries exceeding the predefined threshold of ΔTsk ≥ 0.5 °C in more than one assessment. Thirteen athletes (72.2%) showed recurrent asymmetries in the same anatomical region across multiple assessments (recurrent same-region pattern).

Among athletes with ≥3 assessments, the most frequent region was Adductor-dominant in 5 athletes, Posterior Thigh BF-dominant in 4 athletes and Posterior Thigh ST-dominant in 3 athletes. Consistency (proportion of assessments in which the dominant region was abnormal) ranged from 0.33 to 0.67. Given the modest consistency and the very low ICC, these findings are interpreted as recurrent thermal asymmetry patterns rather than stable thermal phenotypes.

The mean TAB across all assessments was 0.34 ± 0.18 °C (median 0.32 °C, IQR 0.24–0.43 °C, range 0.00–0.97 °C) (Table 5). Notable match-to-match variability was observed using both non-parametric and parametric approaches (Kruskal–Wallis H = 27.53, *p* = 0.025; one-way ANOVA F (15,80) = 2.24, *p* = 0.011), confirming the robustness of this finding. The highest mean TAB values were recorded following matches against Cagliari R (0.55 °C), Cremonese (0.50 °C), and Cesena (0.45 °C), while the lowest values were observed after matches against Lazio (0.18 °C) and Roma (0.19 °C). Match-to-match variation in TAB remained significant after accounting for within-athlete dependence (GEE omnibus Wald test χ^2^ = 315.36, df = 15, *p* < 0.001).

No time-loss muscle injuries were recorded during the entire observation period.

**Table 5:** Thermal Asymmetry Burden (TAB) summary and match-to-match variation.

Parameter	Value
Mean ± SD	0.34 °C ± 0.18 °C
Median (IQR)	0.32 °C (0.24–0.43)
Minimum-Maximum	0.00–0.97 °C
Kruskal–Wallis (by match)	H = 27.53, *p* = 0.025
One-way ANOVA (by match)	F (15,80) = 2.24, *p* = 0.011
ICC (mixed-effects model)	0.024

Note: Highest mean TAB values by match: Cagliari R (0.55 °C), Cremonese (0.50 °C), Cesena (0.45 °C). Lowest mean TAB values by match: Lazio (0.18 °C), Roma (0.19 °C). ICC = Intraclass correlation coefficient; IQR = Interquartile range.

## 4. Discussion

The present longitudinal observational study enabled repeated thermographic assessments throughout an entire competitive season in elite under-18 soccer players using a highly standardized protocol. All thermographic acquisitions were performed 48 h after official matches under controlled environmental conditions and according to established international recommendations for infrared thermography, thereby minimizing measurement variability unrelated to post-match physiological recovery [5,21]. This emphasis on standardized acquisition procedures is further supported by recent evidence demonstrating excellent agreement between automated and manual thermographic analyses when standardized protocols are followed [14].

The principal finding of this study is that thermal asymmetries exceeding the predefined threshold of ΔTsk ≥ 0.5 °C were highly prevalent during standardized 48-h post-match monitoring, occurring in 79.2% of all assessments. The mean maximum ΔTsk per assessment was 0.77 ± 0.43 °C, indicating that localized thermal alterations frequently persisted despite a recovery interval commonly considered sufficient for returning to regular training activities. Linear mixed-effects models accounting for the repeated-measures structure confirmed an overall mean TAB score of 0.358 °C and revealed a very low intraclass correlation coefficient (ICC = 0.024), demonstrating that most variability occurred within athletes across match rather than representing stable differences between individuals.

The very low ICC provides important insight into the biological nature of post-match thermal responses. Rather than reflecting fixed individual thermal characteristics, skin temperature asymmetry appears to represent a dynamic physiological response that varies according to the specific demands imposed by each match. Soccer exposes players to continuously changing combinations of sprinting, accelerations, decelerations, changes in direction, eccentric muscle contractions, and repeated high-intensity actions [1,3,4]. These mechanical loads are associated with localized vascular and metabolic responses [11,20] that continue to evolve during recovery, making transient thermal asymmetries a plausible indicator of recovery status rather than a stable athlete-specific thermal phenotype. This interpretation is further supported by the substantial within-player variability observed across repeated assessments, suggesting that thermographic responses reflect the interaction between acute match load and individual recovery processes.

After accounting for within-athlete dependence, only the Anterior Thigh showed a consistently significant side-to-side difference across analytical approaches (Table 3). The previously observed differences for Adductor and Posterior Thigh BF were no longer statistically significant once clustering was taken into account. More importantly, the anatomical distribution of relevant asymmetries showed a clear predominance of the posterior thigh muscles, particularly the biceps femoris (40.6%) and semitendinosus (35.4%). More importantly, the anatomical distribution of relevant asymmetries showed a clear predominance of the posterior thigh muscles, particularly the biceps femoris (40.6%) and semitendinosus (35.4%). This distribution is consistent with the literature describing the hamstring complex as biomechanically vulnerable during soccer-specific movements requiring repeated eccentric loading at high running speeds, acceleration, and deceleration [1,2,10,11]. According to this literature, hamstring muscles experience substantial mechanical strain during the terminal swing phase of sprinting and explosive directional changes, which has been proposed as a factor prolonging recovery responses. However, as local mechanical load was not directly quantified in the present study, the association between posterior-chain thermal asymmetries and mechanical demand remains a plausible hypothesis rather than a demonstrated mechanism. With this caveat in mind these regions may warrant particular attention in future post-match thermographic monitoring protocols specifically designed to test this hypothesis.

Recent evidence further supports the physiological relevance of monitoring skin temperature throughout the post-match recovery period. Studies have described a characteristic temporal evolution of skin temperature, with marked alterations immediately after competition, persistence during the first 24 h, and recovery that may remain incomplete beyond 48 h depending on exercise intensity and accumulated load [20,22]. In elite football players, skin temperature responses have also been associated with match-induced physical demands and well-being during congested competitive periods [10]. Collectively, these findings indicate that residual thermal asymmetries detected 48 h after competition are compatible with ongoing physiological recovery rather than necessarily indicating pathological tissue damage.

A major strength of the present investigation is its longitudinal repeated-measures design. Unlike most previous studies, which have primarily relied on single post-match evaluations or short monitoring periods [8,15,16,17], our protocol characterized within-athlete thermal behaviour across an entire competitive season. Using predefined operational criteria—an asymmetry of ΔTsk ≥ 0.5 °C present in at least two assessments for recurrent asymmetry, and the same anatomical region exceeding this threshold on at least two occasions for a same-region pattern—recurrent asymmetries were common: fifteen of eighteen athletes (83.3%) exhibited relevant asymmetries on multiple occasions, while thirteen athletes (72.2%) repeatedly presented asymmetries within the same anatomical region. At the same time, the consistency of the dominant anatomical region remained only moderate (0.33–0.67), together with the very low ICC, indicating that recurrent asymmetry patterns coexist with considerable match-to-match variability. These findings suggest that athletes may develop individualized recovery tendencies without exhibiting rigid or permanent thermal signatures. From a physiological perspective, recurrent patterns may reflect preferential loading of specific muscle groups according to individual movement strategies, previous training adaptations, or subtle biomechanical characteristics while remaining sensitive to the specific demands of each competition.

Another noteworthy finding is the significant match-to-match variability observed in TAB scores (Kruskal–Wallis *p* = 0.025; ANOVA *p* = 0.011), with the highest values occurring after specific matches such as Cagliari R, Cremonese, and Cesena. This variability may be related to differences in competitive intensity, tactical demands, cumulative fatigue, and contextual factors known to influence physiological recovery [3,4,10]; however, these variables were not directly quantified in the present study, and their specific contribution to the observed variability remains speculative. Rather than viewing post-match thermography as a binary assessment based solely on the presence or absence of a regional asymmetry, these results suggest that the overall thermal burden may fluctuate substantially according to the characteristics of each match.

In this context, the TAB score represents a potentially useful contribution to longitudinal athlete monitoring. Unlike isolated regional asymmetries, which provide localized information, the TAB score summarizes the overall thermal burden across multiple lower-limb regions into a single quantitative metric. Although exploratory, this composite approach may facilitate longitudinal tracking of recovery status, simplify communication between medical and performance staff, and help identify unusually demanding matches that warrant individualized recovery strategies. Because the TAB score integrates information from several anatomical regions simultaneously, it may better capture the cumulative physiological impact of competition than isolated regional measurements alone. Future studies should determine whether changes in TAB correlate with objective measures of external load, internal load, biochemical recovery markers, and subsequent performance outcomes [3,4].

An additional clinically relevant observation is that no time-loss muscle injuries occurred during the monitoring period despite the high prevalence of relevant thermal asymmetries. While no causal inference can be drawn in the absence of a control group, this finding suggests that recurrent thermal alterations should not automatically be interpreted as indicators of imminent injury. Instead, they may represent physiological adaptations associated with normal recovery from competition. This interpretation aligns with previous literature proposing infrared thermography as a complementary component of athlete monitoring rather than a stand-alone diagnostic or predictive tool for injury prevention [8,19]. The clinical value of thermography may therefore lie less in predicting injuries from single abnormal measurements and more in identifying unusual deviations from an athlete’s typical recovery profile when interpreted alongside other monitoring variables.

Taken together, these findings support a shift from interpreting isolated thermal asymmetries toward evaluating longitudinal response patterns. Rather than relying exclusively on universal thresholds, practitioners may obtain greater clinical value by establishing athlete-specific thermal baselines and monitoring deviations from each player’s typical recovery profile over time [7,21,23]. Repeated longitudinal assessments may therefore help distinguish expected post-match fluctuations from atypical responses that warrant closer clinical observation or individualized recovery interventions. Collectively, these findings extend the current literature by demonstrating that repeated thermographic assessments across an entire competitive season reveal dynamic, individualized recovery patterns that cannot be fully captured through isolated post-match evaluations.

### Limitations

Several limitations should be acknowledged. First, the study involved players from a single elite under-18 soccer team, which may limit the generalizability of the findings to other age groups, competitive levels, or sporting disciplines. Second, the absence of a control group prevents definitive conclusions regarding the effectiveness of the thermography-guided intervention protocol for injury prevention. Third, no time-loss muscle injuries occurred during the observation period. Although this finding may be considered clinically positive, it prevented the evaluation of the predictive value of thermal asymmetries, TAB, or recurrent patterns for subsequent injury occurrence. Fourth, playing position (defender, midfielder or forward) was not recorded in the dataset; therefore position-specific analyses could not be performed. Fifth, the thermal camera used is a general-purpose portable device and was not specifically developed or calibrated for human medical or sports thermography. Although its declared thermal sensitivity is <40 mK (equivalent to 0.04 °C) and its nominal absolute accuracy is ±2 °C, the primary outcome of the present study was bilateral skin-temperature asymmetry (ΔTsk), for which relative side-to-side temperature differences were of primary interest. Consequently, the interpretation of our findings depends more directly on the camera’s ability to reliably detect relative thermal gradients under standardized measurement conditions than on absolute temperature accuracy. Notably, thermal cameras with comparable specifications and the same stated accuracy have been used in published studies of athletes, including the HIKMICRO M60 used by Cabizosu et al. [13] to assess bilateral lower-limb thermal asymmetries. Nevertheless, we acknowledge that potential side-specific and random measurement errors cannot be excluded, and future studies should replicate these findings using cameras specifically validated for human skin thermography.

Sixth, a key methodological limitation is the lack of an age-specific validated thermographic threshold for Under-18 athletes. Adopting the adult threshold ΔTsk ≥ 0.5 °C provides a standardized benchmark, but it should not be misinterpreted as an established clinical biomarker in youth populations. Therefore, the high prevalence of thermal asymmetries observed in this cohort (79.2%) reflects threshold exceedance relative to adult standards rather than confirmed clinical pathology. Future studies are needed to determine whether lower-limb thermal responses in developing athletes require age-specific normative cut-offs.

Seventh, specific statistical limitations regarding cluster modeling should be noted. Generalized estimating equations were used to account for within-athlete dependence; however, no small-sample correction (e.g., Mancl–DeRouen or Kauermann–Carroll) was applied to the robust standard errors. Given the relatively small number of clusters (*n* = 18) and the unbalanced number of observations per athlete (range 1–15), the GEE standard errors should be interpreted with appropriate caution. In addition, the chi-square goodness-of-fit test comparing the prevalence of relevant asymmetries across anatomical regions treated the 96 assessments as independent observations, as a formal mixed-effects logistic approach was not applied.

Additionally, the concept of recurrent thermal asymmetry patterns and the TAB score should currently be considered exploratory. Their reproducibility and potential relationship with training load and injury risk require confirmation in larger prospective cohorts. Finally, although all assessments were performed under highly standardized conditions, thermographic measurements remain susceptible to individual and contextual factors (hydration, sleep, psychological stress, subtle environmental variations). External and internal load metrics were not integrated into the present analysis and should be incorporated in future investigations.

## 5. Conclusions

This prospective longitudinal observational study demonstrates that relevant thermal asymmetries exceeding the predefined threshold of ΔTsk ≥ 0.5 °C are frequently detectable 48 h after official matches in elite under-18 soccer players. The observed thermal responses showed substantial within-athlete and match-to-match variability, as indicated by the very low intraclass correlation coefficient, while recurrent asymmetries were nevertheless observed across several athletes and anatomical regions. These findings support the interpretation of post-match thermal asymmetries as dynamic and recurrent patterns rather than as stable individual thermal phenotypes.

The significant variation in TAB scores across matches further indicates that the overall thermal asymmetry profile may vary substantially between competitive events. However, the present study does not establish the physiological mechanisms underlying these thermal patterns, nor does it demonstrate that thermal asymmetries or TAB scores predict subsequent muscle injury. In the absence of a control group and injury events, infrared thermography should therefore be considered a complementary monitoring tool rather than a diagnostic or predictive method.

From a practical perspective, repeated thermographic assessment may provide useful longitudinal information when interpreted together with clinical evaluation and other athlete-monitoring measures. Establishing individual longitudinal reference profiles, as well as validating age-specific threshold for youth populations, rather than relying exclusively on universal thresholds, may represent a promising approach for future investigation. Larger prospective studies integrating thermographic measurements with external and internal load, recovery indicators, biochemical markers, and injury outcomes are required to determine the reproducibility, physiological relevance, and clinical utility of recurrent thermal asymmetry patterns and the TAB score.

## Figures and Tables

**Table 1:** (**a**) Cohort characteristics and distribution of assessments per athlete. (**b**) Distribution of the number of assessments per athlete.

(**a**)	
**Variable**	**Value**
Players	18
Age range	17–18 years
Playing positions	Defenders, midfielders, forwards
Matches monitored	16
Valid assessments	96
Assessments per athlete (mean ± SD)	5.33 ± 3.65
Assessments per athlete (median)	5
Range of assessments per athlete	1–15
Muscle injuries (time-loss)	0
(**b**)	
**Number of Assessments**	**Number of Athletes**
1	1
2	1
3	6
5	5
6	1
7	1
10	1
12	1
15	1
Total	18

Note: Data are presented as absolute numbers or ranges where appropriate.

**Table 2:** Summary of thermal asymmetry parameters.

Parameter	Value
Assessments with ≥1 ROI ≥ 0.5 °C	76/96 (79.2%)
Mean maximum ΔTsk	0.77 ± 0.43 °C
Median maximum ΔTsk (IQR)	0.70 °C (0.50–1.00)
Range of maximum ΔTsk	0.20–2.40 °C
Assessments with max ΔTsk < 0.5 °C	20 (20.8%)
Assessments with max ΔTsk 0.5–<1.0 °C	49 (51.0%)
Assessments with max ΔTsk 1.0–1.5 °C	22 (22.9%)
Assessments with max ΔTsk > 1.5 °C	5 (5.2%)
Athletes with recurrent asymmetries	15/18 (83.3%)
Athletes with recurrent same-ROI pattern	13/18 (72.2%)

Note: ΔTsk = absolute temperature difference between contralateral regions. IQR = interquartile range. ROI = Region of Interest.

**Table 3:** Side-to-side (Right vs. Left) skin temperature differences according to analytical approach.

Region of Interest	Naïve Paired *t*-Test (n = 96)	Athlete-Level Paired *t*-Test (n = 18)	GEE Exchangeable
	Mean Δ (95% CI), *p*	Mean Δ (95% CI), *p*	Mean Δ (95% CI), *p*
Anterior Thigh	+0.12 (+0.04, +0.20), 0.004	+0.10 (+0.01, +0.18), 0.049	+0–12 (+0.04, +0.20), 0.002
Adductor	−0.09 (−0.18, −0.01), 0.039	−0.06 (−0.15, +0.04), 0.233	−0.09 (−0.19, +0.01), 0.072
Anterior Leg	+0.08 (−0.00, +0.16), 0.054	+0.08 (−0.01, +0.17), 0.106	+0.08 (+0.01, +0.14), 0.024
Posterior Thigh BF	−0.14 (−0.27, −0.01) 0.032	−0.11 (−0.25, +0.03), 0.132	−0.14 (−0.29, +0.01), 0.060
Posterior Thigh ST	−0.04 (−0.15, +0.08), 0.536	+0.02 (−0.13, +0.16), 0.839	−0.04 (−0.20, +0.13), 0.669
Posterior Leg	−0.01 (−0.10, +0.08), 0.799	+0.03 (−0.08, +0.15), 0.588	−0.01 (−0–13, +0.10), 0.844

Note: BF = biceps femoris; ST = semitendinosus.

**Table 4:** Prevalence of relevant asymmetries (ΔTsk ≥ 0.5 °C) across regions.

Region	n/96	%
Anterior Thigh	19	19.8
Adductor	29	30.2
Anterior Leg	16	16.7
Posterior Thigh BF	39	40.6
Posterior Thigh ST	34	35.4
Posterior Leg	20	20.8

Note: Chi-square goodness-of-fit test: x2 = 16.31, *p* = 0.006 (significant differences among regions). BF = biceps femoris region; ST = semitendinosus region.

## Data Availability

The original contributions presented in this study are included in the article. Further inquiries can be directed to the corresponding author.

## Abbreviations

The following abbreviations are used in this manuscript:

ΔTsk	Skin temperature asymmetry
BF	Biceps femoris
ROI	Region of interest
ST	Semitendinosus
TAB	Thermal asymmetry burden

## References

[1] Ekstrand J., Hägglund M., Waldén M. (2011). Injury incidence and injury patterns in professional football: The UEFA injury study. Br. J. Sports Med..

[2] Hägglund M., Waldén M., Ekstrand J. (2006). Previous injury as a risk factor for injury in elite football: A prospective study over two consecutive seasons. Br. J. Sports Med..

[3] Gabbett T.J. (2016). The training–injury prevention paradox: Should athletes be training smarter and harder?. Br. J. Sports Med..

[4] Impellizzeri F.M., Marcora S.M., Coutts A.J. (2019). Internal and external training load: 15 years on. Int. J. Sports Physiol. Perform..

[5] Moreira D.G., Costello J.T., Brito C.J., Adamczyk J.G., Ammer K., Bach A.J.E., Costa C.M.A., Eglin C., Fernandes A.A., Fernández-Cuevas I. (2017). Thermographic imaging in sports and exercise medicine: A Delphi study and consensus statement on the measurement of human skin temperature. J. Therm. Biol..

[6] Ring E.F.J., Ammer K. (2012). Thermography in sports medicine. Physiol. Meas..

[7] Lubkowska A., Chudecka M., Radecka B. (2022). Infrared thermography as a non-invasive method for monitoring physiological responses in athletes. Appl. Sci..

[8] Côrte A.C., Pedrinelli A., Marttos A., Hernandez A.J., Esteves C.D., Figueiredo A.M., Cohen M. (2019). Infrared thermography study as a complementary method of screening and prevention of muscle injuries: Pilot study. BMJ Open Sport Exerc. Med..

[9] Bandeira F., Neves E.B., Moura M.A.M., Nohama P. (2014). The thermography in sports activity: A systematic review. Infrared Phys. Technol..

[10] Huygaerts S., Cos F., Cohen D.D., Calleja-González J., Guitart M., Blazevich A.J., Alcaraz P.E. (2020). Mechanisms of Hamstring Strain Injury: Interactions between Fatigue, Muscle Activation and Function. Sports.

[11] Priego Quesada J.I., Carpes F.P., Bini R.R., Salvador Palmer R., Pérez-Soriano P., Cibrián Ortiz de Anda R.M. (2015). Relationship between skin temperature and muscle activation during incremental cycle exercise. J. Therm. Biol..

[12] Brioschi M., Yeng L., Teixeira M.J. (2015). Medical Thermography: What is it? And Its Applications. Pan Am. J. Med..

[13] Cabizosu A., Ruiz-Angui V., Carazo-Díaz C., Martínez-Noguera F.J., Alcaraz P.E. (2026). Morphological and Thermographic Factors of the Lower Limbs Before Competition and Their Impact on Performance at the Spanish National Cross Country Championships. Biosensors.

[14] Januário W.M., Prímola-Gomes T.N., Rovira-Esteve M., Wanner S.P., Sendra-Pérez C., Priego-Quesada J.I. (2026). Agreement between automated ThermoHuman® and manual thermographic analyses for assessing lower-limb skin temperatures before and after running. J. Therm. Biol..

[15] Marzano-Felisatti J.M., Martínez-Amaya A., Priego-Quesada J.I. (2023). Preliminary analysis of skin temperature asymmetries in elite young tennis players. Appl. Sci..

[16] De León-Muñoz A., Priego-Quesada J.I., Marzano-Felisatti J.M., Sánchez-Jiménez J.L., Sendra-Pérez C., Aparicio-Aparicio I. (2024). Preliminary application of infrared thermography to monitoring of skin temperature asymmetries in professional padel players. Sensors.

[17] del Estal A., Brito C.-J., Galindo V.-E., López Díaz de Durana A., Franchini E., Sillero-Quintana M. (2017). Thermal asymmetries in striking combat sports athletes measured by infrared thermography. Sci. Sports.

[18] Hildebrandt C., Raschner C., Ammer K. (2010). An overview of recent application of medical infrared thermography in sports medicine in Austria. Sensors.

[19] Gómez-Carmona P.M., Pino-Ortega J., Ibáñez S.J., Rico-González M. (2020). Infrared thermography protocol on reducing the incidence of soccer injuries: A systematic review. Int. J. Environ. Res. Public Health.

[20] Rojas-Valverde D., Tomás-Carús P., Timón R., Batalha N., Sánchez-Ureña B., Gutiérrez-Vargas R., Olcina G. (2021). Short-Term Skin Temperature Responses to Endurance Exercise: A Systematic Review of Methods and Future Challenges in the Use of Infrared Thermography. Life.

[21] Fernández-Cuevas I., Marins J.C.B., Lastras J.A., Carmona P.M.G., Cano S.P., García-Concepción M.Á., Sillero-Quintana M. (2015). Classification of factors influencing the use of infrared thermography in humans: A review. Infrared Phys. Technol..

[22] Fernandes A.A., de Assis M.G., Marins J.C.B., de Andrade A.G.P., Albuquerque M.R., Brito C.J., da Silva C.D., do Valle M.A.A.N., Pimenta E.M., Garcia E.S. (2025). Kinetics of skin temperature in lower limbs of professional soccer athletes. Biol. Sport.

[23] Weber V., López D.A., Ochmann D.T., Zentgraf S., Nägele M., Neuberger E.W.I., Schömer E., Simon P., Hillen B. (2026). Deep learning-based infrared thermography reveals reproducible uniform and individual thermoregulatory responses during running. Sci. Rep..

